# 14-Angeloyloxycacalohastine from *Psacalium peltatum*

**DOI:** 10.1107/S1600536812004199

**Published:** 2012-02-10

**Authors:** Nadia Rojano-Vilchis, Simón Hernández-Ortega, Manuel Jimenez-Estrada, Armando Torres Avilez

**Affiliations:** a Instituto de Química, Universidad Nacional Autónoma de México, Circuito Exterior, Ciudad Universitaria, México, DF 04510, Mexico

## Abstract

The title compound [systematic name: (9-meth­oxy-3,5-dimethyl-5,6-dihydro­naphtho­[2,3-*b*]furan-4-yl)methyl 2-meth­yl­but-2-enoate], C_21_H_24_O_4_, was isolated from matarique, or *Psacalium peltatum* (Kunth). The structure is almost planar. The angelo­yloxy group makes an angle of 62.08 (2)° with the furan­oeremophilane skeleton. The carbonyl O atom is disordered between two positions with a 76:24 ratio. The mol­ecules in the crystal are joined by very weak C—H—O inter­actions in the *ac* plane.

## Related literature

For fundamental background information, see: Romo de Vivar *et al.* (2007 ▶). For biological activity, see: Acevedo-Quiroz *et al.* (2008 ▶); Alarcón-Aguilar *et al.* (2000 ▶); Bye *et al.* (1995 ▶); Contreras-Weber *et al.* (2002 ▶); Jimenez-Estrada *et al.* (2006 ▶). For compound isolation, see: Abdo *et al.* (1992 ▶); Bohlmann *et al.* (1977 ▶). For bond-length data, see: Allen *et al.* (1987 ▶).
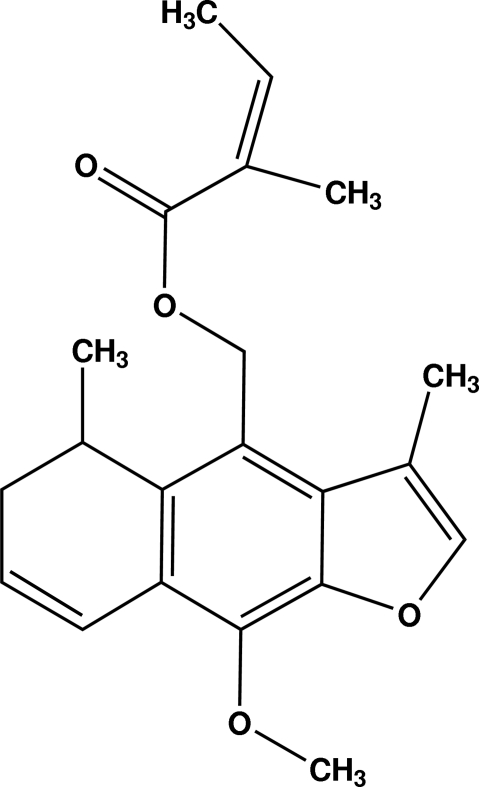


## Experimental

### 

#### Crystal data

C_21_H_24_O_4_
*M* *_r_* = 340.40
Orthorhombic, 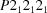

*a* = 7.1627 (17) Å
*b* = 10.276 (2) Å
*c* = 24.605 (6) Å
*V* = 1811.1 (7) Å^3^
*Z* = 4
Mo *K*α radiation
μ = 0.09 mm^−1^
*T* = 298 K
0.40 × 0.40 × 0.40 mm

#### Data collection

Bruker SMART APEX CCD area-detector diffractometer
20061 measured reflections
1945 independent reflections
1701 reflections with *I* > 2σ(*I*)
*R*_int_ = 0.037

#### Refinement

*R*[*F* ^2^ > 2σ(*F* ^2^)] = 0.035
*wR*(*F* ^2^) = 0.095
*S* = 1.12
1945 reflections
241 parameters
21 restraints
H-atom parameters constrained
Δρ_max_ = 0.15 e Å^−3^
Δρ_min_ = −0.15 e Å^−3^

### 

Data collection: *SMART* (Bruker, 1999 ▶); cell refinement: *SAINT* (Bruker, 1999 ▶); data reduction: *SAINT*; program(s) used to solve structure: *SHELXS97* (Sheldrick, 2008 ▶); program(s) used to refine structure: *SHELXL97* (Sheldrick, 2008 ▶); molecular graphics: *SHELXTL* (Sheldrick, 2008 ▶); software used to prepare material for publication: *SHELXTL*.

## Supplementary Material

Crystal structure: contains datablock(s) I, global. DOI: 10.1107/S1600536812004199/aa2037sup1.cif

Structure factors: contains datablock(s) I. DOI: 10.1107/S1600536812004199/aa2037Isup2.hkl

Supplementary material file. DOI: 10.1107/S1600536812004199/aa2037Isup3.cml

Additional supplementary materials:  crystallographic information; 3D view; checkCIF report

## Figures and Tables

**Table 1:** Hydrogen-bond geometry (Å, °)

*D*—H⋯*A*	*D*—H	H⋯*A*	*D*⋯*A*	*D*—H⋯*A*
C15—H15*A*⋯O2^i^	0.97	2.62	3.536 (3)	157
C6—H6*B*⋯O4^ii^	0.97	2.61	3.42 (2)	142

Symmetry codes: (i) 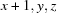; (ii) 
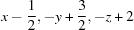
.

**Fig. 1.**
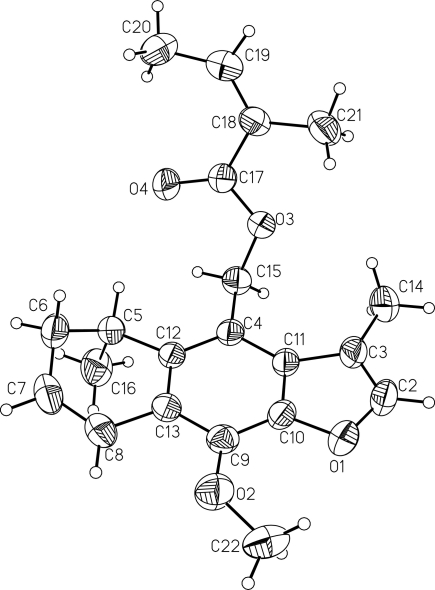
The structure of I with the numbering scheme. The thermal ellipsoids are drawn at 40% probability level. The disordered O4A atom was omitted for clarity.

## supplementary crystallographic information

### Comment

The sesquiterpenes knows as eremophilanes contain in its basis skeleton a decalin system and most of them are found as furanoeremophilanes (Romo de Vivar *et al.*, 2007). *Psacalium peltatum* (Kunth) Cass., is an endemic medicinal plant, a member of matarique complex, widely distributed in the central part of Mexico. The roots of *P. peltatum* have been shown biological activities (Alarcón-Aguilar *et al.*, 2000; Bye *et al.*, 1995; Contreras-Weber *et al.*, 2002). Sesquiterpenes as cacalol and cacalone, isolated from *P. decompositum*, have been shown a clear inhibition of edema with a dose dependent in anti-inflammatory effect using *in vivo* models (Jimenez-Estrada *et al.*, 2006). Even more, cacalone in a natural mixture with *epi*-cacalone reported the highest anti-inflammatory effect using *in vivo* 12-*O*-tetradecanoylphorbol-13-acetate (TPA) model (Acevedo-Quiroz, *et al.*, 2008). Although the title compound has been isolated from several species of *Senecio inaequidens*, *S. othonnae* (Bohlmann *et al.*, 1977) and *S. canescens* (Abdo *et al.*, 1992), no report on the crystal structure determination of this compound has appeared. Therefore, due to this lack of data, the *x*-ray crystal structure determination of 14-angeloyloxycacalohastine was made.

14-Angeloyloxycacalohastine (I) has a furanoeremophilane skeleton (Fig. 1). Bond lengths and angles in (I) exhibit normal values (Allen *et al.*, 1987). The structure is almost planar with C6 and C7 atoms out of the plane, forming a dihedral angle of 26.9 (1)° between central benzene ring and C4—C5—C6—C7 atoms. The angeloyloxy frame is almost perpendicular making a dihedral angle of 62.08 (2) to the furanoeremophilane skeleton. In absence of donor H atoms is noteworthy the fact that in the crystal structure, the molecules are linked by weak C—H···O intermolecular interaction (Table 1).

### Experimental

Roots of *Psacalium peltatum* (Kunth) Cass., were collected from pine-oak forest of Mineral del Chico, Hidalgo, Mexico]. A voucher specimen was deposited at the National Herbarium (MEXU 1138692) of the Instituto of Biologia, UNAM, Mexico. Air-dried and powdered roots of *P. peltatum* were sequentially extracted with n-hexane by exhaustive maceration (3 × 2 l), at room temperature. Hexane extract of roots from *P. peltatum*, was separated in a chromatoghraphic column by elution with hexane - ethyl acetate in gradient mixture. 14-Angeloyloxycacalohastine was isolated from the fraction eluted by hexane.

### Refinement

The positional parameters of H atoms were calculated geometrically (C—H = 0.93–0.98 Å). All H atoms were refined as riding with U_iso_(H) = 1.5U_eq_(C) for methyl H-atoms and U_iso_(H) = 1.2U_eq_(C) for other H-atoms. The carbonyl oxygen is disordered and has been refined in two positions. The ratio of SOF is 76/24 for O4/O4A respectively. In absence of heavy atoms the absolute configuration was not determined and the Friedel pairs were merged.

### Crystal data

**Table:**

C_21_H_24_O_4_	*F*(000) = 728
*M**_r_* = 340.40	*D*_x_ = 1.248 Mg m^−^^3^
Orthorhombic, *P*2_1_2_1_2_1_	Mo *K*α radiation, λ = 0.71073 Å
Hall symbol: P 2ac 2ab	Cell parameters from 9953 reflections
*a* = 7.1627 (17) Å	θ = 2.6–25.1°
*b* = 10.276 (2) Å	µ = 0.09 mm^−^^1^
*c* = 24.605 (6) Å	*T* = 298 K
*V* = 1811.1 (7) Å^3^	Prism, colourless
*Z* = 4	0.40 × 0.40 × 0.40 mm

### Data collection

**Table:**

Bruker SMART APEX CCD area-detector diffractometer	1701 reflections with *I* > 2σ(*I*)
Radiation source: fine-focus sealed tube	*R*_int_ = 0.037
Graphite monochromator	θ_max_ = 25.4°, θ_min_ = 2.2°
Detector resolution: 0.83 pixels mm^-1^	*h* = −8→8
ω scans	*k* = −12→12
20061 measured reflections	*l* = −29→29
1945 independent reflections	

### Refinement

**Table:**

Refinement on *F*^2^	Primary atom site location: structure-invariant direct methods
Least-squares matrix: full	Secondary atom site location: difference Fourier map
*R*[*F*^2^ > 2σ(*F*^2^)] = 0.035	Hydrogen site location: inferred from neighbouring sites
*wR*(*F*^2^) = 0.095	H-atom parameters constrained
*S* = 1.12	*w* = 1/[σ^2^(*F*_o_^2^) + (0.0598*P*)^2^] where *P* = (*F*_o_^2^ + 2*F*_c_^2^)/3
1945 reflections	(Δ/σ)_max_ = 0.001
241 parameters	Δρ_max_ = 0.15 e Å^−^^3^
21 restraints	Δρ_min_ = −0.15 e Å^−^^3^

### Special details

**Table:**

Geometry. All s.u.'s (except the s.u. in the dihedral angle between two l.s. planes) are estimated using the full covariance matrix. The cell s.u.'s are taken into account individually in the estimation of s.u.'s in distances, angles and torsion angles; correlations between s.u.'s in cell parameters are only used when they are defined by crystal symmetry. An approximate (isotropic) treatment of cell s.u.'s is used for estimating s.u.'s involving l.s. planes.
Refinement. Refinement of *F*^2^ against ALL reflections. The weighted *R*-factor *wR* and goodness of fit *S* are based on *F*^2^, conventional *R*-factors *R* are based on *F*, with *F* set to zero for negative *F*^2^. The threshold expression of *F*^2^ > σ(*F*^2^) is used only for calculating *R*-factors(gt) *etc*. and is not relevant to the choice of reflections for refinement. *R*-factors based on *F*^2^ are statistically about twice as large as those based on *F*, and *R*- factors based on ALL data will be even larger.

### Fractional atomic coordinates and isotropic or equivalent isotropic displacement parameters (Å^2^)

**Table:**

	*x*	*y*	*z*	*U*_iso_*/*U*_eq_	Occ. (<1)
O1	0.2416 (2)	0.31693 (16)	0.75432 (6)	0.0714 (5)	
O2	−0.0374 (2)	0.52184 (18)	0.78161 (7)	0.0781 (5)	
O3	0.6537 (2)	0.35107 (13)	0.92670 (5)	0.0575 (4)	
C2	0.4000 (4)	0.2430 (2)	0.75866 (9)	0.0744 (7)	
H2	0.4239	0.1717	0.7364	0.089*	
C3	0.5167 (3)	0.2821 (2)	0.79734 (9)	0.0609 (6)	
C4	0.4777 (3)	0.48417 (18)	0.86340 (7)	0.0474 (5)	
C5	0.3937 (4)	0.6883 (2)	0.91708 (8)	0.0600 (6)	
H5	0.4798	0.6518	0.9440	0.072*	
C6	0.2181 (4)	0.7334 (2)	0.94676 (10)	0.0741 (7)	
H6A	0.1798	0.6668	0.9723	0.089*	
H6B	0.2463	0.8117	0.9672	0.089*	
C7	0.0626 (4)	0.7604 (2)	0.90877 (11)	0.0724 (7)	
H7	−0.0248	0.8239	0.9176	0.087*	
C8	0.0457 (3)	0.6957 (2)	0.86251 (10)	0.0618 (6)	
H8	−0.0526	0.7154	0.8392	0.074*	
C9	0.1341 (3)	0.5079 (2)	0.80519 (8)	0.0529 (5)	
C10	0.2624 (3)	0.4122 (2)	0.79325 (7)	0.0521 (5)	
C11	0.4302 (3)	0.39708 (18)	0.82102 (7)	0.0487 (5)	
C12	0.3504 (3)	0.58296 (18)	0.87563 (7)	0.0464 (5)	
C13	0.1786 (3)	0.59388 (19)	0.84724 (7)	0.0500 (5)	
C14	0.6933 (4)	0.2148 (3)	0.81219 (10)	0.0830 (8)	
H14A	0.7065	0.1375	0.7907	0.124*	
H14B	0.6903	0.1919	0.8500	0.124*	
H14C	0.7971	0.2717	0.8055	0.124*	
C15	0.6592 (3)	0.4671 (2)	0.89264 (8)	0.0561 (5)	
H15A	0.7597	0.4593	0.8664	0.067*	
H15B	0.6836	0.5429	0.9150	0.067*	
C16	0.4913 (4)	0.8027 (2)	0.88907 (12)	0.0783 (8)	
H16A	0.4144	0.8343	0.8600	0.117*	
H16B	0.6091	0.7743	0.8747	0.117*	
H16C	0.5116	0.8712	0.9150	0.117*	
C17	0.6327 (3)	0.3665 (2)	0.97976 (9)	0.0557 (5)	
O4	0.5953 (19)	0.4714 (3)	0.99988 (18)	0.074 (2)	0.76 (3)
O4A	0.697 (5)	0.4664 (12)	0.9991 (6)	0.071 (4)	0.24 (3)
C18	0.6328 (3)	0.2407 (2)	1.00916 (8)	0.0562 (5)	
C19	0.6373 (3)	0.2364 (2)	1.06292 (10)	0.0677 (6)	
H19	0.6366	0.1532	1.0778	0.081*	
C20	0.6433 (5)	0.3438 (3)	1.10315 (9)	0.0836 (8)	
H20A	0.5185	0.3719	1.1111	0.125*	
H20B	0.7133	0.4153	1.0885	0.125*	
H20C	0.7021	0.3138	1.1359	0.125*	
C21	0.6300 (4)	0.1180 (2)	0.97545 (11)	0.0767 (7)	
H21A	0.6139	0.0440	0.9988	0.115*	
H21B	0.7457	0.1099	0.9561	0.115*	
H21C	0.5285	0.1219	0.9500	0.115*	
C22	−0.0716 (4)	0.4709 (3)	0.72916 (10)	0.0860 (8)	
H22A	−0.0871	0.3783	0.7314	0.129*	
H22B	0.0322	0.4906	0.7059	0.129*	
H22C	−0.1830	0.5093	0.7146	0.129*	

### Atomic displacement parameters (Å^2^)

**Table:**

	*U*^11^	*U*^22^	*U*^33^	*U*^12^	*U*^13^	*U*^23^
O1	0.0921 (12)	0.0633 (10)	0.0588 (9)	0.0009 (10)	−0.0122 (9)	−0.0173 (8)
O2	0.0667 (10)	0.0908 (12)	0.0767 (11)	0.0047 (10)	−0.0186 (9)	−0.0119 (10)
O3	0.0778 (9)	0.0467 (7)	0.0479 (7)	0.0093 (8)	−0.0067 (8)	0.0009 (6)
C2	0.111 (2)	0.0586 (14)	0.0541 (13)	0.0083 (16)	0.0060 (14)	−0.0168 (11)
C3	0.0824 (15)	0.0526 (12)	0.0477 (11)	0.0086 (12)	0.0124 (11)	−0.0027 (10)
C4	0.0607 (12)	0.0424 (10)	0.0390 (10)	−0.0013 (10)	0.0045 (9)	0.0044 (8)
C5	0.0849 (16)	0.0449 (11)	0.0501 (11)	0.0098 (12)	−0.0100 (11)	−0.0052 (9)
C6	0.115 (2)	0.0514 (13)	0.0554 (12)	0.0123 (14)	0.0105 (13)	−0.0075 (11)
C7	0.0786 (16)	0.0509 (13)	0.0878 (18)	0.0144 (14)	0.0141 (14)	−0.0071 (13)
C8	0.0660 (14)	0.0470 (11)	0.0724 (14)	0.0065 (12)	0.0023 (12)	0.0059 (11)
C9	0.0608 (12)	0.0493 (11)	0.0486 (10)	−0.0041 (11)	−0.0022 (10)	0.0068 (9)
C10	0.0702 (13)	0.0453 (11)	0.0407 (9)	−0.0046 (11)	0.0000 (10)	−0.0025 (9)
C11	0.0669 (12)	0.0402 (10)	0.0389 (9)	0.0001 (10)	0.0074 (9)	0.0027 (8)
C12	0.0636 (12)	0.0372 (9)	0.0385 (9)	−0.0001 (10)	0.0013 (9)	0.0035 (8)
C13	0.0648 (12)	0.0396 (10)	0.0455 (10)	−0.0014 (10)	0.0036 (10)	0.0069 (8)
C14	0.101 (2)	0.0752 (17)	0.0732 (16)	0.0340 (16)	0.0106 (15)	−0.0099 (13)
C15	0.0649 (12)	0.0520 (12)	0.0514 (11)	0.0023 (12)	0.0032 (10)	0.0045 (10)
C16	0.0901 (18)	0.0530 (13)	0.0918 (18)	−0.0111 (14)	−0.0088 (15)	−0.0155 (13)
C17	0.0678 (13)	0.0483 (12)	0.0509 (11)	0.0002 (11)	−0.0043 (11)	−0.0024 (9)
O4	0.123 (6)	0.0456 (13)	0.0548 (15)	0.0098 (19)	0.006 (2)	−0.0040 (11)
O4A	0.105 (11)	0.050 (4)	0.059 (5)	0.003 (6)	−0.020 (6)	−0.009 (4)
C18	0.0589 (13)	0.0493 (11)	0.0603 (13)	0.0018 (11)	−0.0077 (10)	0.0068 (10)
C19	0.0669 (14)	0.0678 (14)	0.0685 (14)	0.0039 (13)	−0.0034 (12)	0.0160 (12)
C20	0.099 (2)	0.0994 (19)	0.0529 (13)	0.0107 (19)	−0.0019 (14)	0.0008 (13)
C21	0.0969 (19)	0.0458 (13)	0.0873 (16)	0.0053 (13)	−0.0173 (16)	0.0029 (12)
C22	0.0881 (18)	0.105 (2)	0.0646 (14)	−0.0133 (19)	−0.0220 (14)	0.0098 (14)

### Geometric parameters (Å, º)

**Table:**

O1—C2	1.370 (3)	C10—C11	1.392 (3)
O1—C10	1.378 (2)	C12—C13	1.420 (3)
O2—C9	1.366 (3)	C14—H14A	0.9600
O2—C22	1.414 (3)	C14—H14B	0.9600
O3—C17	1.324 (2)	C14—H14C	0.9600
O3—C15	1.458 (2)	C15—H15A	0.9700
C2—C3	1.329 (3)	C15—H15B	0.9700
C2—H2	0.9300	C16—H16A	0.9600
C3—C11	1.456 (3)	C16—H16B	0.9600
C3—C14	1.487 (4)	C16—H16C	0.9600
C4—C12	1.397 (3)	C17—O4	1.216 (3)
C4—C11	1.415 (3)	C17—O4A	1.221 (8)
C4—C15	1.497 (3)	C17—C18	1.481 (3)
C5—C12	1.519 (3)	C18—C19	1.324 (3)
C5—C6	1.527 (4)	C18—C21	1.510 (3)
C5—C16	1.532 (4)	C19—C20	1.483 (4)
C5—H5	0.9800	C19—H19	0.9300
C6—C7	1.480 (4)	C20—H20A	0.9600
C6—H6A	0.9700	C20—H20B	0.9600
C6—H6B	0.9700	C20—H20C	0.9600
C7—C8	1.324 (3)	C21—H21A	0.9600
C7—H7	0.9300	C21—H21B	0.9600
C8—C13	1.463 (3)	C21—H21C	0.9600
C8—H8	0.9300	C22—H22A	0.9600
C9—C10	1.377 (3)	C22—H22B	0.9600
C9—C13	1.397 (3)	C22—H22C	0.9600
			
C2—O1—C10	104.50 (17)	C3—C14—H14B	109.5
C9—O2—C22	120.3 (2)	H14A—C14—H14B	109.5
C17—O3—C15	118.19 (16)	C3—C14—H14C	109.5
C3—C2—O1	114.2 (2)	H14A—C14—H14C	109.5
C3—C2—H2	122.9	H14B—C14—H14C	109.5
O1—C2—H2	122.9	O3—C15—C4	110.43 (18)
C2—C3—C11	105.3 (2)	O3—C15—H15A	109.6
C2—C3—C14	124.8 (2)	C4—C15—H15A	109.6
C11—C3—C14	129.9 (2)	O3—C15—H15B	109.6
C12—C4—C11	117.49 (19)	C4—C15—H15B	109.6
C12—C4—C15	123.27 (18)	H15A—C15—H15B	108.1
C11—C4—C15	119.24 (19)	C5—C16—H16A	109.5
C12—C5—C6	111.7 (2)	C5—C16—H16B	109.5
C12—C5—C16	109.76 (18)	H16A—C16—H16B	109.5
C6—C5—C16	111.0 (2)	C5—C16—H16C	109.5
C12—C5—H5	108.1	H16A—C16—H16C	109.5
C6—C5—H5	108.1	H16B—C16—H16C	109.5
C16—C5—H5	108.1	O4—C17—O3	122.2 (3)
C7—C6—C5	112.01 (19)	O4A—C17—O3	116.2 (10)
C7—C6—H6A	109.2	O4—C17—C18	125.1 (3)
C5—C6—H6A	109.2	O4A—C17—C18	122.9 (6)
C7—C6—H6B	109.2	O3—C17—C18	112.17 (19)
C5—C6—H6B	109.2	C19—C18—C17	121.1 (2)
H6A—C6—H6B	107.9	C19—C18—C21	121.5 (2)
C8—C7—C6	121.2 (2)	C17—C18—C21	117.43 (18)
C8—C7—H7	119.4	C18—C19—C20	130.0 (2)
C6—C7—H7	119.4	C18—C19—H19	115.0
C7—C8—C13	121.4 (2)	C20—C19—H19	115.0
C7—C8—H8	119.3	C19—C20—H20A	109.5
C13—C8—H8	119.3	C19—C20—H20B	109.5
O2—C9—C10	125.7 (2)	H20A—C20—H20B	109.5
O2—C9—C13	116.9 (2)	C19—C20—H20C	109.5
C10—C9—C13	117.22 (19)	H20A—C20—H20C	109.5
O1—C10—C9	125.70 (19)	H20B—C20—H20C	109.5
O1—C10—C11	110.82 (18)	C18—C21—H21A	109.5
C9—C10—C11	123.45 (19)	C18—C21—H21B	109.5
C10—C11—C4	119.90 (19)	H21A—C21—H21B	109.5
C10—C11—C3	105.17 (19)	C18—C21—H21C	109.5
C4—C11—C3	134.9 (2)	H21A—C21—H21C	109.5
C4—C12—C13	121.13 (17)	H21B—C21—H21C	109.5
C4—C12—C5	121.96 (19)	O2—C22—H22A	109.5
C13—C12—C5	116.80 (18)	O2—C22—H22B	109.5
C9—C13—C12	120.79 (19)	H22A—C22—H22B	109.5
C9—C13—C8	119.6 (2)	O2—C22—H22C	109.5
C12—C13—C8	119.60 (18)	H22A—C22—H22C	109.5
C3—C14—H14A	109.5	H22B—C22—H22C	109.5

### Hydrogen-bond geometry (Å, º)

**Table:**

*D*—H···*A*	*D*—H	H···*A*	*D*···*A*	*D*—H···*A*
C15—H15*A*···O2^i^	0.97	2.62	3.536 (3)	157
C6—H6*B*···O4^ii^	0.97	2.61	3.42	142

Symmetry codes: (i) *x*+1, *y*, *z*; (ii) *x*−1/2, −*y*+3/2, −*z*+2.
